# Patients with chronic kidney disease are not well adhered to dietary recommendations: a cross-sectional study

**DOI:** 10.1186/s40795-020-00333-y

**Published:** 2020-04-09

**Authors:** Gebretsadkan Gebremedhin Gebretsadik, Zelalem Debebe Mengistu, Birhanu Worku Molla, Helen Tkuwab Desta

**Affiliations:** 1 Department of Nutrition and Dietetics, School of Public Health, College of Health Sciences, Mekelle University, Mekelle, Ethiopia; 2 Center for Food Science and Nutrition, College of Natural and Computational Sciences, Addis Ababa University, Addis Ababa, Ethiopia; 3 Department of Nephrology, Saint Paul’s Hospital Millennium Medical College, Addis Ababa, Ethiopia

**Keywords:** Dietary intake, Dietary recommendations, CKD, Dietary protein, Dietary energy, Ethiopia

## Abstract

**Background:**

Nutrition and dietary patterns are modifiable factors that can be utilized to prevent or slow the progression of Chronic kidney disease (CKD). Adherence to current dietary recommendations can reduce the incidence, or slow the progression of CKD and reduce mortality. The objectives of this study was to compare the dietary nutrient intake of CKD patients (CKD above stage 3 including hemodialysis) to dietary recommendations and to assess the correlations of those dietary nutrient intakes with each other and with chosen laboratory measurements.

**Methods:**

A hospital-based cross-sectional study was conducted among 100 patients with CKD. A consecutive 7 days dietary record supplemented with interviews for data completion was used to assess dietary intake. Recent clinical laboratory measurements were obtained from patients’ medical records. The obtained dietary data were analyzed by the Ethiopian food composition database and the nutrisurvey software. Dietary energy and nutrients intake were compared with recommendations for CKD patients.

**Results:**

The dietary energy intake (DEI) of almost all patients was below recommended levels. The average Dietary protein intake (DPI) was above the recommended levels (0.95 ± 0.27 g/kg/day) for about 60% of the respondents. Besides, 38% and only two of the respondents had their dietary phosphorus and potassium intakes above recommended levels, respectively. Estimated Glomerular filtration rate (eGFR) was positively correlated with both total and animal protein intakes while blood levels of creatinine and urea were negatively correlated with animal protein intake.

**Conclusion:**

Patients with CKD are not well adhered to dietary recommendations and some nutrients showed correlation with chosen clinical laboratory measurements. Besides, DEI and DPI were below and above recommended levels, respectively, for most patients. Besides, more than one-third of the participants had phosphorus intakes above recommendation. These non-optimal dietary nutrient intakes may contribute to fast clinical deterioration and mortality.

## Background

Recently, the prevalence of CKD has increased exponentially and is costing developed countries a huge proportion of health care finances, while significantly contributing to morbidity, mortality, and decreased life expectancy in the developing ones [1]. The prevalence of CKD among diabetic patients in southern Ethiopia was 18.2 and 23.8% when defined according to the Modification of diet in renal disease (MDRD) and Cockcroft-Gault eq. (C-G) equations, respectively [2].

Dietary therapy has historically been used in the management of CKD and adherence to current dietary guidelines can reduce the incidence, or slow the progression of CKD and reduce mortality [3]. General goals of dietetic management in CKD patients include preserving kidney function, maintaining optimal nutritional status, reducing uremic symptoms, postponing the need for dialysis and aiming for a healthy initiation of dialysis (for Predialysis patients), correcting metabolic imbalances, preventing complications, and improving quality of life and patient-centered outcomes [4, 5].

Steiber found that less than a quarter of the patients with chronic renal failure met 75% or more of their energy and protein needs as patients in different stages were involved in the study [6]. In addition, Duenhas et al. concluded that energy and protein intake in a group of predialysis individuals was too low (24.9 ± 8.2 kcal/kg/day and 0.95 ± 0.36 g/kg/day respectively) [7].

The World health organization (WHO) recommends 0.83 g of protein/Kg of body weight as the safe level for healthy adults [8]. Low protein diet (LPD), which contains 0.8 g (or 0.6 g) of Protein/Kg of body weight according to the recommendations, had been proven to delay the progression of CKD by lengthening the predialysis phase and to reduce renal causes of mortality [9]. In practice, however, patients following this LPD often reduce the amount of consumed food, which creates difficulties in achieving the right balance of energy and other nutrients and makes it very difficult to prevent protein catabolism and subsequent protein-energy malnutrition [10].

In Ethiopia, CKD patients receive dietary advice from physicians and nurses because there is a lack of trained dietitians. Although there are some studies that assessed the prevalence of CKD, none tried to assess the adherence of these patients to dietary recommendations. Therefore, the aim of this study is to compare the dietary nutrient intakes of patients with CKD to recommendations and to assess the correlations of these nutrient intakes swith chosen clinical laboratory measurements.

## Methods

### Study setting, design, and sample size

This hospital-based cross-sectional study was carried out from January to March 2018 in two selected hospitals (St. Paul’s hospital millennium medical college (SPHMMC) and Sante medical center) with CKD outpatient clinics. Data were collected from purposely taken 100 CKD outpatients (patients on follow-up) (including hemodialysis patients) who were selected from both hospitals proportional to their total CKD population (68 participants from SPHMMC and 32 participants from santé medical center).

#### Inclusion criteria

CKD Patients in 3rd – 5th stage with eGFR< 60 ml/min/1.73 m2, patients without previous dietary intervention received from a dietitian/nutritionist, and patients diagnosed at least 6 months before the study were included.

#### Exclusion criteria

Patients diagnosed less than 6 months before the study, those with speaking difficulties, and those unwilling to participate were excluded.

At every follow-up encounter, patients are seen by their doctors and nurses. Patients who came for follow up on the data collection period and met the criteria were approached and included if they give consent.

### Data collection procedures and quality improvement

Sociodemographic data were collected using structured interviewer-administered questionnaire. Weight was measured by a scale with a maximum capacity of 150 kg and accuracy of 0.1 kg and was calibrated after each measurement. Height was measured in standing position in stadiometer but for those confined to bed arm span was used. Body mass index (BMI) was calculated using weight and height.

Biochemical data including eGFR, Creatinine, Urea, phosphate, and other chosen biological indicators (blood levels) were obtained from patients’ medical records. The last three (or two) measurements were obtained from the medical charts and an average was taken as final measurement.

A consecutive seven-day dietary record was obtained from each participant. Before data collection started, participants were instructed about the principles of completing the dietary record, the necessity for an accurate and thorough recording of all food products and beverages consumed, and were provided with food picture models to help them estimate portion sizes. Participants were interviewed at the time of submission of the dietary records to get more complete dietary intake data.

Training of the data collectors, translation of the data collection tool into the local language (Amharic), and pretest of the tool in 10% of the participants were done before data collection started to ensure the quality of the data.

### Statistical analysis

The Ethiopian food composition table and nutrisurvey software were used to analyze the dietary data, and SPSS version 20 was utilized for further statistical analysis.

Energy and nutrients intake of participants were compared to recommendations for CKD patients [11, 12] or to recommendations for healthy individuals when they were not established [8] (Table 1). Recommended Daily Allowance (RDA) was used to assess the adequacy of nutrients, and Adequate Intake (AI) level was used for those nutrients without an established RDA.

**Table 1:** Dietary energy and nutrients recommendations for CKD patients

Nutrients	Recommended intake
Energy [Kcal (KJ)/Kg of body weight]^a, b^	146 (35) – For age below 60
	126–146 (30–35) – For age above 60
Total Protein [g/Kg of body weight]	0.6^1^/0.8^c^ –Predialysis
	1.1–1.2^a^-Dialysis
Animal protein [% of total protein intake]	≥50
Fat[% of energy]^a^	25–35
Carbohydrate [% of energy]^a^	45–60
Fiber [g]^a^	20–30
Sodium [mg]1	2400
Potassium [mg]^a, c^	4700
Phosphorus [mg]	800–1000- For Predialysis patients^a^
	1000–1400- For Dialysis patients^b^
Iron [mg]^c^	8 - For men
	18– For women 19–50
	8 – For women > 50
Vitamin A [μg]^c^	900- for Men
	700- For women
Vitamin D [μg]^a, c^	5- For age 19–50 years
	10-for age 51–70 years
	15- For age above 70 years

^a^Kopple, J. D. (2001) [12]. National kidney foundation K/DOQI clinical practice guidelines for nutrition in chronic renal failure. American Journal of Kidney Diseases, 37(1), S66-S70

^b^James, G., & Jackson, H. (2003) [11]. European guidelines for the nutritional care of adult renal patients. EDTNA/ERCA Journal, 29(1), 23–43

^c^WHO, J. (2007) [8]. Protein and amino acid requirements in human nutrition. World health organization technical report series (935), 1

Mean ± standard deviation for normally distributed data and Median ± Interquartile range for non-normally distributed data are used to present findings. Shapiro-Wilk test was used to check the distribution of the analyzed variables. In order to characterize correlations among factors, Pearson’s and Spearman’s correlation were utilized for normal and non-normal distribution, respectively.

## Results

In this study, for which the response rate was 100%, among the total 100 respondents, the majority (79%) were males. The age of the respondents averaged 47.76 ± 12.98 years, and most (96) were less than 70 years of age. The Sociodemographic characteristics of the respondents are shown below (Table 2).

**Table 2:** Sociodemographic characteristics of CKD patients in two selected hospitals in Addis Ababa, Ethiopia, 2018

Characteristics		Frequency
Sex	Male	79 (79%)
	Female	21 (21%)
Age	< 40	30 (30%)
	41–49	23 (23%)
	50–59	25 (25%)
	60–69	18 (18%)
	> = 70	4 (4%)
Residence	Urban	90 (90%)
	Rural	10 (10%)
Religion	Orthodox	63 (63%)
	Muslim	29 (29%)
	Protestant	7 (7%)
	Catholic	1 (1%)
Marital status	Single	29 (29%)
	Married/living together	60 (60%)
	Divorced	8 (8%)
	Widowed	3 (3%)
Educational level	No formal education	7 (7%)
	Primary	15 (15%)
	Secondary	28 (28%)
	More than secondary	50 (50%)
Occupation	Professional/technical	32 (32%)
	Clerical	8 (8%)
	Sales and services	38 (38%)
	Skilled manual	1 (1%)
	Unskilled manual	2 (2%)
	Agriculture	5 (5%)
	Other	14 (14%)

The findings confirmed that 90 (90%) of the respondents were on dialysis, and 10 (10%) were not on dialysis. About 59(59%) and 39 (39%) were hypertensive and diabetic, respectively.

The average BMI of the respondents was 22.91 ± 3.46 kg/m^2^. The average creatinine, eGFR, and urea levels were 2.9 ± 1.87 mg/dl, 33.4 ± 13.04 ml/min/1.73 m2, and 68.3 ± 22.21 mg/dl respectively (Table 3).

**Table 3:** Anthropometric and laboratory measurements of CKD patients in two selected hospitals in Addis Ababa, Ethiopia, 2018

Characteristics	Mean	SD
Age [Years]	47.76	12.98
Weight [Kg]	64.73	11.31
Height [m]	1.67	0.06
BMI [Kg/m2]	22.91	3.46
Creatinine [mg/dl]	2.9	1.87
GFR [ml/min/1.73 m2]	33.4	13.04
Urea [mg/dl]	68.3	22.21
Potassium [mg/dl]	4.6	0.59
Hemoglobin [mg/dl]	10.6	0.81
Albumin [mg/dl]	3.4	0.35
Phosphate [mM/l]	4.6	1.02

The average DEI was 1394.62 ± 212.61 Kcal (5835.07 ± 889.57 KJ)/day. The energy intake per body weight averaged 22.15 ± 5.01 Kcal/Kg. The average total, animal, and plant protein values of the diet were 0.95 ± 0.27 g/kg, 0.45 ± 0.19 g/kg, and 0.5 ± 0.12 g/kg of body weight, respectively (Table 4).

**Table 4:** Daily average energy value and nutrients content of the diet of CKD patients in two selected hospitals in Addis Ababa, Ethiopia, 2018

Nutrients	Mean	SD
Energy [Kcal (KJ)]	1394.62 (5835.07)	212.61 (889.57)
Energy [Kcal/kg]	22.15	5.01
Carbohydrate [g]	243.99	45.65
Total protein [g]	59.75	12.81
Animal protein [g]	28.12	10.24
Plant protein [g]	31.62	5.70
Protein per weight [g/kg]	0.95	0.27
Fat [g]	32.172	8.45
Sodium [mg]	1925.5	644.15
Potassium [mg]	1854.5	364.34
Phosphorus [mg]	947.86	209.46
Iron [mg]	45.56	12.15
Vitamin D [μg]	1.48	0.58
Fluid [ml]	1121.1	(218.2–2121.4)^a^
Fibre [g]	13.59	5.39

^a^in case of nonparametric variables – median, minimum and maximum values are presented

Carbohydrates provided the majority of the DEI, (65.15 ± 6.7%) ranging 50–79%. The findings confirmed that almost all respondents had their daily energy intakes below recommended levels (only 3 respondents fulfilled the recommendation). The other interesting finding is that 62 (62%) respondents had their total protein intakes on or above recommended levels with the animal protein intake lower than the recommendations in about two-thirds (64%) of the analyzed respondents (Table 5).

**Table 5:** Daily energy and nutrients intake compared to recommendations, of CKD patients in two selected hospitals in Addis Ababa, Ethiopia, 2018

	% of respondents characterized by intake		
	Below recommended level	On recommended level	Above recommended level
Energy	97	1	2
Carbohydrate	0	27	73
Fat	87	13	0
Protein	38	2	60
Animal protein	64	36	0
Sodium	66	20^a^	14
Potassium	98	0^a^	2
Phosphorus	26	36	38
Iron	0	0^a^	100
Vitamin D	100	0^a^	0
Fibre	87	13	0

^a^recommended value±10%

Average DEI was strongly positively correlated with DPI (r = + 0.74, *p* < 0.001). DPI was also positively correlated with animal protein, plant protein, and phosphorus intakes (Table 6).

**Table 6:** Correlation of total, animal, and plant protein intake with Energy and nutrients content of the diet of CKD patients in two selected hospitals in Addis Ababa, Ethiopia, 2018

Nutrients	Total protein		Animal protein		Plant protein	
	R	P	R	P	R	P
Energy	0.737**	0.000	0.577**	0.000	0.602**	0.000
Carbohydrate	0.309**	0.002	−0.145	0.150	0.444**	0.000
Fat	0.489**	0.000	0.596**	0.000	0.110	0.275
Total Protein	–	–	0.574**	0.000	0.627**	0.000
Animal protein	0.574**	0.000	–	–	0.315**	0.001
Plant protein	0.627**	0.000	0.315**	0.001	–	–
Fibre	0.406**	0.000	0.221*	0.027	0.552**	0.000
Sodium	0.050	0.625	−0.142	0.158	0.258**	0.01
Potassium	0.370**	0.000	0.168	0.095	0.535**	0.000
Phosphorus	0.741**	0.000	0.652**	0.000	0.202*	0.044
Iron	−0.097	0.339	−0.244*	0.014	0.170	0.091
Vitamin D	−0.068	0.498	−0.126	0.212	0.075	0.457

**Correlation statistically significant at P ≤ 0.01

*Correlation statistically significant at *P* ≤ 0.05

eGFR was positively weakly correlated with both total (r = + 0.236, *p* = 0.018) and animal (r = + 0.30, *p* = 0.002) protein intakes, but not with plant protein intake. Blood levels of creatinine and urea had a weak negative correlation with animal protein intake (r = − 0.265, *p* = 0.008 and r = − 0.242, *p* = 0.015, respectively) but no correlation with total and plant protein intakes. Besides, blood levels of phosphate showed no correlation with protein and phosphorus intake. (Table 7).

**Table 7:** Correlation of creatinine, eGFR, Urea, blood phosphate, and albumin levels with Energy and nutrients content of the diet of CKD patient in two selected hospitals in Addis Ababa, Ethiopia, 2018

	Energy		Total protein		Animal protein		Plant protein		Phosphorus	
	R	P	R	p	R	P	R	P	r	P
Creatinine	−.039	0.698	−0.169	0.094	−0.265**	0.008	0.097	0.337	0.035	0.731
eGFR	0.160	0.112	0.236*	0.018	0.301**	0.002	0.001	0.994	0.075	0.456
Urea	−0.087	0.392	−0.191	0.057	−0.242*	0.015	−0.005	0.961	−0.088	0.384
Phosphate	0.169	0.093	−0.027	0.792	−0.140	0.164	0.194	0.053	0.149	0.140
Albumin	0.136	0.182	0.222*	0.028	0.215*	0.033	0.154	0.129	0.114	0.265

**Correlation statistically significant at *P* ≤ 0.01

* Correlation statistically significant at *P* ≤ 0.05

## Discussion

The findings of this study confirmed that almost all respondents had their daily energy intakes below recommended levels (only 3 respondents fulfilled the recommendation). Similarly, in other studies, only two individuals met the daily dietary energy recommendations [10] and only 15% of patients reached 75% of their energy requirements [6]. Additionally, Shahar *et al*. found that the mean caloric intake (1683.9+/_546.9 kcal/day) for Haemodialysis (HD) patients deviated almost 20% below guidelines [13]. These similarities might be justified by the fact that all CKD patients have problems getting the right amount of dietary energy due to various factors including reduction in appetite.The average total protein intake in this presented research was 0.95 g/kg of body weight. This is higher than the average DPI in another study in which it was 0.85 g/kg of ideal body weight [10]. This may be reasoned out by the fact that the respondents in the later study were only predialysis patients, who usually limit their protein intake more than patients on dialysis.

Around 60% of the respondents in this study had their total protein intakes above recommended levels. This is a little bit different from a study done in Taiwan in which DPIs were significantly higher than the recommended levels in less than half of the respondents (47.2%) [14]. This variation could be because respondents in this presented research have never received counseling from a dietitian but a subset of those in the Chen et al.*,* study received counseling from registered dietitians in the hospital they attend. Moreover, this difference can be attributed to the fact that protein intake goals set by the dietitians in the hospital, which were different from those set by National kidney foundation/Kidney disease outcomes quality initiative (NKF/KDOQI) intake goals, were used because the dietitians considered the patient’s current intake status.

About two-thirds (64%) of the analyzed respondents had animal protein intakes lower than the recommendations. However, it is recommended that greater than half of the protein intake should be of a high biologic value such as proteins in eggs, fish, poultry, meat, and dairy products because of the presence of essential amino acids [15]. Nevertheless, higher animal protein consumption, including red meat [16], is associated with rendering the kidneys to excrete a higher acid load as compared to higher dietary plant protein intake [17].

In this presented research, only two respondents had their daily dietary potassium intakes above recommendations. In a 5 years cohort study, a higher dietary potassium intake was associated with increased death risk in HD patients, even after adjustments for other nutrient intakes [18]. Conversely, during earlier stages of CKD a diet high in potassium, which is usually low in sodium, may slow the progression of the disease by lowering blood pressure [19]. A small study suggested that the dietary approach to stop hypertension (DASH) diet may be a valuable, non-pharmacologic strategy for blood pressure control in individuals with CKD. However, this needs to be confirmed in larger sample size in order to be recommended [20].

In this presented study, respondents consuming less protein were simultaneously characterized by a lower intake of energy and most other nutrients. Similarly, in other research, CKD patients in highest baseline DPI and DEI quartiles had 4.11-fold (95% confidence interval: 2.79–6.05) higher odds of having protein energy wasting syndrome at month 12 [21]. These similarities may happen as a result of patients limiting the quantity of all consumed foods in an attempt to limit the level of protein in the diet. It is important that for HD patients an adequate energy intake is required to achieve positive nitrogen balance and individualized advice on suitable dietary sources of protein should be delivered by dietitian/nutrition advisors [11, 15].

Respondents with higher protein intake also had higher dietary phosphorus intake. Similarly, a review showed that there is a correlation between dietary intakes of protein and phosphorus [22]. In this study, however, DPI (or dietary phosphorus) was not correlated with serum phosphate levels. This is supported by another study which described that a high protein (and high phosphorus) diet does not always correlate with increased serum phosphate levels [23]. The difference in bioavailability of phosphorus from foods items may be the reason for this. Consuming a greater amount of protein from plants foods and reducing taking foods containing the inorganic phosphate which is readily absorbable and highly contributes to dietary phosphate load will be beneficial in the CKD population [24].

In this presented study, eGFR was significantly positively correlated with both total and animal protein intakes, but not with plant protein intake. Blood levels of creatinine and urea were also significantly negatively correlated with animal protein intake but not with total or plant protein intake. This is supported by other studies. A spontaneous decrease in energy and protein intake followed a decline in renal function in patients with no previous dietary intervention [7]. Similarly, patients with more advanced disease were characterized by significantly lower protein intake, which was associated with limiting animal protein [10]. These similarities in the findings imply the fact that patients reduce animal products, not plant products, which they think make a large source of protein. This may lead to a deficiency of essential amino acids and the risk of acquiring malnutrition. In contrast, other studies showed that lower energy and higher protein intakes than recommended levels may be associated with lowering renal function markers [14, 25] [26]. These studies mostly involved advanced CKD patients and support the current nutrition practice guidelines on providing adequate energy and optimal protein.

This study has some limitations. First, there may be a possibility of selection bias as the 100 study participants were selected purposely (non-random sampling). This was done in order to get an extensive dietary intake data (7 days dietary record) from each patient. However, this might have created problems with the generalizability of the findings, though. Second, since data were collected at one point in time, this study can’t show any cause and effect relationship between variables. In order to draw more reliable findings and conclusions, similar studies with stronger methodologies should be done in the future. Third, Dietary records might not provide an exact estimation of dietary nutrient intake. This is because respondents might have problems with accurately quantifying food portions and some might have changed their eating patterns which might distort the findings and lead to invalid conclusions. This limitation would be overcome if future researchers apply a more objective and stronger dietary intake assessment method to get accurate nutrient intake data and plausible findings.

## Conclusion

Patients with Chronic kidney disease are not well adhered to current dietary recommendations. Respondents consuming less total, animal, or plant protein were simultaneously characterized by lower intake of energy and most other nutrients. Additionally, eGFR was correlated with both total and animal protein intakes, whereas blood levels of creatinine and urea were significantly negatively correlated with animal protein intake but not with total protein, plant protein, or phosphorus intakes. The findings imply that non-optimal dietary nutrient intake may contribute to fast clinical deterioration and mortality in CKD patients. Finally, the findings imply that there is an urgent need of the deployment of dietitians in hospitals that can provide appropriate counselling for patients with CKD.

## Data Availability

The datasets used and/or analyzed during the current study are available from the corresponding author on reasonable request.

## Abbreviations

AI: Adequate intake
BMI: Body mass index
CG: Cockcroft Gault
CKD: Chronic kidney disease
DEI: Dietary energy intake
Dl: Decilitre
DPI: Dietary protein intake
eGFR: Estimated glomerular filtration rate
G/Kg: Gram/Kilogram
IRB: Institutional review board
Kcal: Kilo calories
KDOQI: Kidney disease outcomes quality initiatives
KJ: Kilo joules
LPD: Low protein diet
MDRD: Modification of diet in renal disease
Mg: Milligrams
NKF: National kidney foundation
RDA: Recommended daily allowance
SPHMMC: Saint Paul’s Hospital Millenium Medical College
SPSS: Statistical package for social sciences
WHO: World health organization
